# Serum concentrations of clarithromycin and rifampicin in pulmonary *Mycobacterium avium* complex disease: long-term changes due to drug interactions and their association with clinical outcomes

**DOI:** 10.1186/s40780-015-0029-0

**Published:** 2015-11-14

**Authors:** Hitoshi Shimomura, Sena Andachi, Takahiro Aono, Akira Kigure, Yosuke Yamamoto, Atsushi Miyajima, Takashi Hirota, Keiko Imanaka, Toru Majima, Hidenori Masuyama, Koichiro Tatsumi, Takao Aoyama

**Affiliations:** Faculty of Pharmaceutical Sciences, Tokyo University of Science, 2641 Yamazaki, Noda, Chiba 278-8510 Japan; Department of Pharmacy, Chemotherapy Research Institute, Kaken Hospital, 6-1–14 Konodai, Ichikawa, Chiba 272-0827 Japan; Department of Respiratory medicine, Chemotherapy Research Institute, Kaken Hospital, 6-1–14 Konodai, Ichikawa, Chiba 272-0827 Japan; Department of Respirology, Graduate School of Medicine, Chiba University, 1-8–1 Inohana, Chuo–ku, Chiba 260-8670 Japan

**Keywords:** *Mycobacterium avium* complex, Rifampicin, Clarithromycin, Serum concentration, CYP3A4, 6 beta-hydroxycortisol to cortisol ratio, Clinical efficacy

## Abstract

**Background:**

Concomitant use of clarithromycin (CAM) and rifampicin (RFP) for the treatment of pulmonary *Mycobacterium avium* complex (MAC) disease affects the systemic concentrations of both drugs due to CYP3A4–related interactions. To date, however, there has been no report that investigates the long–term relationship between the drug concentrations, CYP3A4 activity, and clinical outcomes. Our aim was to investigate the time course of the drug levels in long–term treatment of subjects with pulmonary MAC disease, and examine the correlation of these concentrations with CYP3A4 activity and clinical outcomes.

**Methods:**

Urine and blood samples from nine outpatients with pulmonary MAC disease were collected on days 1, 15, and 29 (for four subjects, sample collections were continued on days 57, 85, 113, 141, 169, 225, 281, 337, and 365). Serum drug concentrations and urinary levels of endogenous cortisol (F) and 6 beta-hydroxycortisol (6βOHF), the metabolite of F by CYP3A4, were measured, and evaluated 6βOHF/F ratio as a CYP3A4 activity marker. In addition, the clinical outcomes of 4 subjects were evaluated based on examination of sputum cultures and chest images.

**Results:**

The mean 6βOHF/F ratio increased from 2.63 ± 0.85 (*n* = 9) on the first day to 6.96 ± 1.35 on day 15 and maintained a level more than double initial value thereafter. The serum CAM concentration decreased dramatically from an initial 2.28 ± 0.61 μg/mL to 0.73 ± 0.23 μg/mL on day 15. In contrast, the serum concentration of 14-hydroxy-CAM (M-5), the major metabolite of CAM, increased 2.4-fold by day 15. Thereafter, both CAM and M-5 concentrations remained constant until day 365. The explanation for the low levels of serum CAM in pulmonary MAC disease patients is that RFP-mediated CYP3A4 induction reached a maximum by day 15 and remained high thereafter. Sputum cultures of three of four subjects converted to negative, but relapse occurred in all three cases.

**Conclusions:**

Our study demonstrated that serum CAM concentrations in pulmonary MAC disease patients were continuously low because of RFP-mediated CYP3A4 induction, which may be responsible for the unsatisfactory clinical outcomes.

## Background

*Mycobacterium avium* complex (MAC) is the most common etiologic agent of lung disease caused by nontuberculous mycobacteria [1]. Because MAC is not susceptible to antituberculous drugs, clarithromycin (CAM) is the key drug for treatment of pulmonary MAC disease, and multidrug therapy with rifampicin (RFP) and ethambutol (EB) is recommended to prevent the development of resistant bacteria [2, 3].

RFP induces metabolic enzymes such as CYP3A4 and, as a result, decreases the serum or plasma concentration of concomitantly administered CAM and promotes its metabolism to 14-hydroxy-CAM (M-5), which is less effective against MAC than the parent compound [4–6]. Yamamoto and colleagues [4] reported that the mean plasma CAM concentration on the 8^th^ day after RFP addition decreased to 20 %. Taki and colleagues [5] demonstrated that the mean serum CAM concentration decreased to 15 % on the 3^rd^ day after RFP addition. Wallace and colleagues [6] reported that the plasma CAM concentration decreased to 13 % when concomitantly administered with RFP (mean medication period: 4.4 months). RFP-mediated enzyme induction begins within 2–4 days after starting administration, reaches a maximum within 6–10 days, and returns to preinduction levels 2–3 weeks after stopping the administration of the drug [7]. In contrast, administration of CAM increases the serum or plasma concentrations of any concomitantly administered drugs by inhibiting p-glycoprotein and CYP3A4 [8, 9]. Previous reports have demonstrated that the plasma concentration of RFP increased when RFP was administered together with CAM [10]. In humans, the main metabolic pathway of RFP is deacetylation to 25-desacetylrifampicin (DR) by arylacetamide deacetylase [11].

When both RFP and CAM are used together to treat pulmonary MAC, low serum CAM concentrations may result in an unfavorable treatment outcome and the emergence of resistant bacteria. In addition, side effects such as liver damage, leukopenia, or thrombocytopenia can occur when serum concentrations of RFP rise. According to the current guidelines provided by the American Thoracic Society, Japanese Society for Tuberculosis (JSTB), and Japanese Respiratory Society (JRS), the chemotherapeutic treatment period for pulmonary nontuberculous mycobacteria (NTM) disease is approximately one year after culture-negative conversion [12, 13]. Previous studies investigating the serum or plasma concentrations of CAM and RFP in patients with pulmonary MAC disease were limited to short-term studies focused on the early period of treatment, around 1 week [4, 5], or examined results at only one time point after long-term treatment [6]. Koh and colleagues [14] claimed that there was no association between low plasma CAM concentrations in patients with pulmonary MAC disease and treatment outcomes. However, only one sample was collected for each patient in their study. Therefore, although the therapy needed to be long term, it remained unclear whether the exposure level to drugs was constant during the therapy. Furthermore, there has been no report that investigates the long-term relationship between drug concentrations and CYP3A4 activity, together with an examination of efficacy.

The present study was designed to monitor the long-term time course of drug concentrations in the serum of subjects with pulmonary MAC disease treated with RFP and CAM. We also measured the urinary concentrations of endogenous cortisol (F) and 6 beta-hydroxycortisol (6βOHF), the metabolite of F by CYP3A4, and evaluated the 6βOHF/F ratio as a marker of the activity of CYP3A4. Furthermore, we evaluated the clinical outcomes of patients who continued this study for 1 year based on the examination of sputum cultures and chest images.

## Methods

### Subjects

Nine outpatients diagnosed with pulmonary MAC disease at the Chemotherapy research institute, Kaken hospital (Kaken hospital) from 2012 to 2014 were enrolled in this study. Patients under 20 years of age, over 80 years of age, or with hepatic or renal dysfunction were excluded. All patients were required to fulfill the JSTB and JRS criteria. Doses of CAM, RFP, and EB were based on the recommendations of both societies. Urine and blood samples were collected on days 1, 15, 29 (for four subjects, sample collections were continued on days 57, 85, 113, 141, 169, 225, 281, 337, and 365). Urine samples were collected on the morning of the first day before a medical examination and the subjects were administered the drugs after lunch with blood collected two hours later. On days 15, 29, 57, 85, 113, 141, 169, 225, 281, 337, and 365, the subjects took the drugs at home after breakfast and then two hours later, urine and blood samples were collected before a medical examination in the hospital. The urine samples were collected at almost the same time as on the first day. Serum and urine samples were frozen and stored at –80 °C until analysis.

This study was conducted in compliance with the ethical guidelines for clinical studies. It was approved by the Ethical Review Committee at Tokyo University of Science (approval and study number: 12001) and Kaken hospital (approval and study number: 9) and registered in the University hospital Medical Information Network-Clinical Trial Registry (November 15, 2012, ID: UMIN000009343). All patients provided written informed consent before entry into the study.

### Reagents

The internal standard roxithromycin, CAM, RFP, DR, and F were purchased from Wako Pure Chemical Industries, Ltd. (Osaka, Japan), while 6βOHF and prednisolone, which served as an internal standard for analysis, were purchased from SIGMA-Aldrich Japan Co. (Tokyo, Japan). M-5 was kindly provided by Taisho Toyama Pharmaceutical Co., Ltd. (Tokyo, Japan).

### Measurement of serum drug concentrations and urinary 6βOHF and F concentrations

The serum concentrations of CAM, M-5, RFP, and DR were measured using the liquid chromatography-tandem mass spectrometry method described by Oswald et al. [15], with modifications. The liquid chromatography-tandem mass spectrometry system used consisted of an LC-20 AD high-performance liquid chromatography (HPLC) system (Shimadzu Co., Kyoto, Japan) coupled with an API 3200 mass spectrometer (AB Sciex Japan, Ltd., Tokyo, Japan). The separations were performed on the reverse phase column Atrantis T3 (5 μm, 2.1 × 150 mm, Nihon Waters Co. Ltd., Tokyo, Japan) and using the solvent mixtures (A) 10 mM ammonium acetate–acetonitrile (20:80) and (B) 10 mM ammonium acetate-acetonitrile (80:20) with the following gradient: 40 % B increased to 85 % B over 4 min, decreased to 40 % B over 1.25 min, increased to 85 % B over 0.85 min, and equilibrated back to 40 % B over 0.9 min.

Urinary 6βOHF and F concentrations were measured using the HPLC-UV method described by Hu et al. [16], with modifications. The HPLC system used consisted of an L-2130 pump and an L-2455 UV detector (HITACHI High-Tech Science Co. Ltd., Tokyo, Japan). The detection wavelength was fixed 245 nm. The separations were performed on the reverse phase column PEGASIL ODS SP100 (5 μm, 4.6 × 250 mm, Senshu Scientific Co., Ltd., Tokyo, Japan), and using the solvent mixtures (A) acetonitrile and (B) acetonitrile-water (17.5: 82.5), with the gradient starting at 100 % B and decreasing to 63.6 % B over 80 min.

### Statistical analyses

The serum concentration for each drug and the ratio of urinary 6βOHF/F are presented as the mean ± standard error (SE). Data on day 15 were compared with each initial value using paired Student’s t-test during the initial period of study (the day 1–29). The significance level for each comparison was set as *P* <0.05. Serum RFP concentrations (mean ± SE) were plotted against the ratio of urinary 6βOHF/F (mean ± SE) from the same time point and the relationship between the values was examined using the Pearson correlation coefficient. All statistical analyses were performed using Excel 2010 (Microsoft Co., Redmond, WA, USA).

### Clinical outcomes

The clinical outcomes of four subjects who continued in this study for 1 year were evaluated by examination of sputum cultures and chest images at the indicated time points. The cultures were scored as “– (negative)”, “1+”, “2+”, “3+”, or “4+”. Culture negative conversion was defined as three consecutive negative cultures; with the time of conversion defined as the date of the first negative culture. The subjects were monitored and relapse was recorded during the observation period (until day 365) and post-observation period (after day 365), as the first day of a positive culture after negative scores. Chest images were evaluated by a pulmonologist, and classified as “Improved”, “Unchanged”, and “Worsened” compared to the previous assessment.

### CAM susceptibility test

CAM susceptibility tests were performed using the minimum inhibitory concentration (MIC) method for 3 of the 4 subjects who continued in this study for 1 year. If sputum culture conversion was not achieved after 169 days, isolates from the last positive cultures collected were tested. According to the JSTB and JRS guidelines for chemotherapy of pulmonary nontuberculous mycobacterial disease-2012 revised version [13], strains with MIC of CAM (≦4 μg/mL) were considered susceptible to CAM, while those with an MIC ≧32 μg/mL were considered resistant. Values of 8–16 μg/mL were considered intermediate.

## Results

### Subject characteristics and dosage

Subject characteristics and dosage are summarized in Table 1. Etiologic agents were identified as *M. avium* in seven subjects, *M. intracellulare* in one subject, and *M. avium*-*M. intracellulare* overlapping in one subject. The subject population comprised two male and seven female subjects. The averages of age and body weight (mean ± SE) were 66.6 ± 2.4 years old and 50.7 ± 2.4 kg, respectively. The daily dose of drugs consisted of CAM 600–800 mg, RFP 450–600 mg, and EB 500–750 mg. There were no subjects who had the history of premedication with CAM before diagnosis as pulmonary MAC disease.

**Table 1:** Details of subjects with pulmonary *Mycobacterium avium* complex disease and dosage of each drug

Subject	Gender	Age (years)	Weight (kg)	Species	Dosage (mg/day)		
					CAM	RFP	EB
A	female	68	58	*M.avium*	800	600	750
B	male	70	64	*M.avium*	800	450	750
C	female	68	43	*M.avium*	600	450	500
D	female	64	42	*M.avium*	800	450	500
E	female	50	51	*M.intracellulare*	800	450	750
F	female	63	47	*M.avium*	800	450	750
G	female	70	56	*M.avium*	800	600	750
H	female	71	48	*M.avium*	800	450	750
I	male	75	47	*M.avium* and *M.intracellulare*	800	450	750

### Time course of the ratio of urinary 6βOHF/F and the serum concentrations of drugs for the initial period of the study (days 1–29)

**Fig. 1:**
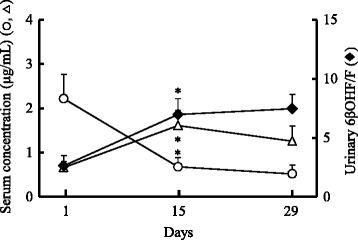
Relationship between CYP3A4 activity and serum clarithromycin or its metabolite concentrations for the initial period. Time course of serum concentrations of clarithromycin (CAM, ○), 14-hydroxy-CAM (M-5, △), and urinary 6β-hydroxycortisol/cortisol (6βOHF/F, ♦) ratio up to day 29 (*n* = 9, mean ± SE). *P <0.05 compared with each initial value

**Fig. 2:**
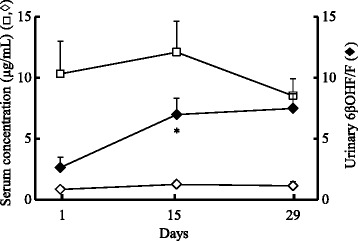
Relationship between CYP3A4 activity and serum rifampicin or its metabolite concentrations for the initial period. Time course of serum concentrations of rifampicin (RFP, □) and 25-desacetyl-RFP (DR, ◊), and the ratio of urinary 6β-hydroxycortisol/cortisol (6βOHF/F, ♦) up to day 29 (*n* = 9, mean ± SE). *P < 0.05 compared with each initial value

The mean ratio of 6βOHF/F, a marker for activity of CYP3A4, significantly increased from 2.63 ± 0.85 (*n* = 9) on the first day, to 6.96 ± 1.35 (2.6-fold) on day 15, and 7.46 ± 1.21 (2.8-fold) on day 29 (Fig. 1). The concentration of CAM in the serum significantly decreased to approximately 30 % of the initial value, from 2.28 ± 0.61 μg/mL on the first day to 0.73 ± 0.23 μg/mL on day 15, and did not notably decrease thereafter (day 29: 0.55 ± 0.22 μg/mL). The concentration of M-5 in the serum increased 2.4-fold, from 0.69 ± 0.15 μg/mL on the first day to 1.66 ± 0.38 μg/mL on day 15, and 1.9-fold on day 29 (1.28 ± 0.38 μg/mL). Serum RFP concentrations did not alter (around 10 μg/mL) up to day 29, and the concentrations of DR were approximately one-tenth of the RFP concentration (Fig. 2).

### Time course of the ratio of urinary 6βOHF/F and the concentrations of drugs in the serum in the long-term (days 1–365)

**Fig. 3:**
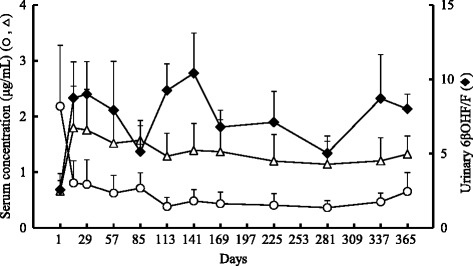
Long-term changes of CYP3A4 activity and serum clarithromycin or its metabolite concentrations. Time course of serum concentration of clarithromycin (CAM, ○) and 14-hydroxy-CAM (M-5, △), and the ratio of urinary 6β-hydroxycortisol/cortisol (6βOHF/F, ♦) up to the day 365 (*n* = 4, mean ± SE)

**Fig. 4:**
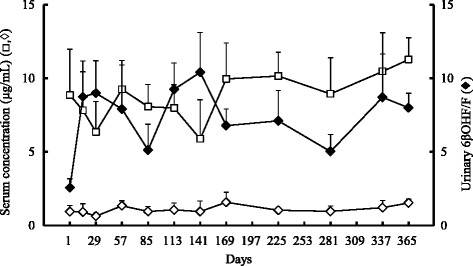
Long-term changes of CYP3A4 activity and serum rifampicin or its metabolite concentrations. Time course of serum concentration of rifampicin (RFP, □) and 25-desacetyl-RFP (DR, ◊), and ratio of urinary 6β-hydroxycortisol/cortisol (6βOHF/F, ♦) up to day 365 (*n* = 4, mean ± SE)

**Fig. 5:**
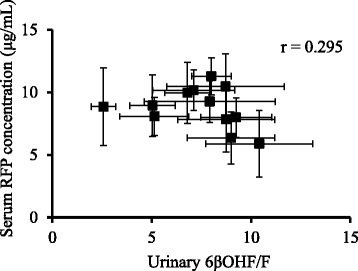
Relationship between CYP3A4 activity and serum rifampicin concentration in the long-term. Relationships between the ratio of urinary 6β-hydroxycortisol/cortisol (6βOHF/F) and serum concentrations of rifampicin (RFP). Symbols represent values on days 1 to 365 (*n* = 4, mean ± SE)

The mean 6βOHF/F ratio of four subjects who continued in this study for 1 year increased from 2.57 ± 0.61 on the first day to 3.4 times this amount on day 15, and maintained values more than double the initial value thereafter (Fig. 3). The concentration of CAM in the serum decreased dramatically to approximately 40 % of the initial value, from 2.18 ± 1.10 μg/mL on the first day to 0.80 ± 0.40 μg/mL on day 15, and remained constant until day 365. Serum M-5 concentration increased 2.7-fold, from 0.66 ± 0.31 μg/mL on the first day to 1.80 ± 0.75 μg/mL on day 15, and maintained consistently high values from day 29 to the end of the study. The mean of serum RFP concentrations were in the range from 5.88 to 11.3 μg/mL (Fig. 4). At each point, the serum DR concentration was approximately one-tenth of the RFP concentration. The relationship between the ratio of urinary 6βOHF/F and serum RFP concentrations was poor (*r* = – 0.295, Fig. 5).

### Clinical outcomes by examination of sputum cultures and chest images

The sputum cultures of three out of four subjects converted to negative (subject B; day 29, subject C; day 57, and subject E; day 225), whereas the cultures of one subject remained positive (Table 2). Relapse, however, occurred in all the three subjects with negative sputum cultures (subject C; on day 225, and subjects B and E; after day 365). The chest images of the 4 subjects rarely worsened, but generally showed a slight improvement.

**Table 2:** Clinical efficacy of treatment for pulmonary *Mycobacterium avium* complex patients

Subject	Evaluation	Follow-up period (Days)											MIC/(CAM) (μg/mL)	Post follow-up period
		1	29	57	85	113	141	169	225	281	337	365		Remarks
B	Sputum culture	1+	– ^a^	–	–	–	–	–	–	–	–	–	No data	Relapsed at day 553
	Chest image		Unchanged	Unchanged	Unchanged	Improved	Unchanged	Unchanged	Unchanged	Unchanged	Unchanged	Improved		
C	Sputum culture	1+	1+	– ^a^	–	–	–	No data	1+ ^b^	–	1+	1+	0.06	
	Chest image		Unchanged	Unchanged	Improved	Unchanged	Unchanged	Unchanged	Unchanged	Unchanged	Unchanged	Unchanged	(Day 365)	
D	Sputum culture	1+	1+	1+	–	1+	1+	1+	1+	1+	1+	1+	0.25	
	Chest image		Unchanged	Unchanged	Improved	Unchanged	Unchanged	Unchanged	Unchanged	Unchanged	Unchanged	Unchanged	(Day 365)	
E	Sputum culture	–	1+	–	1+	–	1+	1+	– ^a^	–	–	–	0.06	Relapsed at day 589
	Chest image		Improved	Unchanged	Improved	Unchanged	Worsened	Improved	Unchanged	Unchanged	Unchanged	Unchanged	(Day 169)	

MIC; Minimal inhibitory concentrations, CAM; Clarithromycin

^a^Culture negative conversion: the first day of three consecutive negative cultures

^b^Relapse: the first day of a positive culture after negative culture conversion

### CAM susceptibility test

The MIC values are shown in Table 2. CAM-resistant MAC isolates were not detected in the subjects who were examined using the CAM susceptibility test in this study.

## Discussion

This is the first study to investigate the long-term changes in the serum concentrations of CAM, RFP, and their metabolites in pulmonary MAC disease patients and to examine the correlation of these concentrations with the ratio of urinary 6βOHF/F and clinical outcomes.

Our results in the initial stage of treatment (days 1–29) showed a remarkable decrease in serum CAM concentrations with a change from baseline in accordance with an earlier report [4, 5]. This study succeeded in clarifying that CAM concentration in the serum decreased significantly by day 15, whereas the ratio of 6βOHF/F and serum M-5 concentration increased over the same period of time (Fig. 1). This was not measured in the previous studies. This increased metabolism from CAM to M-5 resulted from the induction of the enzyme, CYP3A4 by RFP. Although there was a 2-to 4-fold variation in the mean of 6βOHF/F over the extended period of the study (days 1–365), after day 15, the decrease in serum CAM concentrations and the increase in serum M-5 concentrations were maintained consistently throughout the study period (Fig. 3). These findings suggest that serum CAM and M-5 concentrations were maintained at their new levels if the induction of CYP3A4 reached a certain point (more than double of the initial value in this study).

On the other hand, CYP3A4 hardly affects the metabolism of RFP because the main metabolic pathway of RFP is deacetylation to 25-desacetylrifampicin (DR) by arylacetamide deacetylase [11]. In subjects with tuberculosis, receiving daily doses of RFP, the serum RFP concentrations increased after the first dose, decreased during the first month, and remained constant without a significant decrease thereafter [17, 18]. Serum RFP concentrations were reportedly lower in patients with tuberculosis who received repeated RFP administrations than that in healthy adults or in patients receiving this therapy for the first time. It was suggested that these results were a consequence of RFP-mediated induction of the enzyme involved in the biliary excretion [17]. In the present study however, all the subjects were diagnosed with pulmonary MAC disease, and all of them received CAM in addition to RFP. CAM is known to behave as a motilin agonist with gastrointestinal motor-stimulating activity resulting in an increase in gastric emptying rate [19], thereby antagonizing the effect of p-glycoprotein in the gastrointestinal tract or kidney [8] and CYP3A4 in the intestine or liver [9]. These effects of CAM are thought to increase the serum concentration of concomitantly administered drugs. Therefore, these effects might have led to the partial alteration of serum RFP concentrations in this study.

Alffenaar and colleagues [10] collected plasma samples at nine points per day eight weeks after the beginning of the therapy and demonstrated that total RFP exposure increased in patients receiving CAM compared to that in patients receiving RFP without CAM (1.2-fold in maximum drug concentration and 1.6-fold in area under the plasma concentration curve). They speculated that CAM inhibitory effects on the p-glycoprotein-mediated efflux of RFP were responsible. Although serum RFP concentration increased on some observation day in our study, we could not calculate the maximum drug concentration and area under the concentration curve because serum RFP concentration was measured only once on each examination day in this study. Therefore, we were not able to compare our results with theirs. However, interestingly, the serum RFP concentration and urinary 6βOHF/F ratio often intersected with each other. When the serum RFP concentration was high, the urinary 6βOHF/F ratio was low, and vice versa. We examined the relationship between the ratio of urinary 6βOHF/F and serum RFP concentrations and found a poor correlation (Fig. 5), suggesting that the serum RFP concentration was not directly affected by the level of CYP3A4 induction. Since it was reported that 450 mg of RFP a day achieved maximum induction of enzyme [20], the dose of RFP administered to subjects in this study should have been sufficient for maximum induction of CYP3A4. Accordingly, even if the serum RFP concentrations varied to some extent, RFP-mediated CYP3A4 induction would have been expected to reach the maximum by day 15 and remain high, explaining the consistently low serum CAM concentrations in the study subjects with pulmonary MAC disease despite the administration of recommended standard treatment.

Currently, the bacilli negative conversion rates of the standard treatment for pulmonary MAC disease have been reported to be approximately 60–80 % [21–25], with relapse occurring in 48 % of patients after completion of treatment [26]. Therefore, this disease is often difficult to treat. In our study, the sputum cultures of three out of four subjects converted to negative, but relapse occurred in all three cases (Table 2). All three subjects whose sputum culture conversion was not achieved after 169 days (subject C, D, and E) remained susceptible to CAM on the testing day. The decreased CAM concentrations in each patient were generally higher than MICs for MAC isolated from each patient. However, predicting the clinical efficacy by direct comparison with the drug concentration is difficult because MIC means the minimal drug concentration to inhibit growth of bacteria in vitro. MAC originally colonizes the airways, and then infects the epithelial cells and macrophages [27], suggesting that the concentrations of antimicrobials in epithelial lining fluid (ELF) in lungs are an important factor in its suppression. The CAM concentration in the extracellular fluid to exhibit the efficacy against MAC has been reported to be 1–8 μg/mL [5, 28–30]. Hasegawa and colleagues [30] demonstrated that the ELF/serum CAM concentration ratio in patients treated with 400 or 800 mg/day of CAM was highly individual and ranged between 2.0 and 41.5. Although they concluded that 800 mg/day of CAM administration was an appropriate dose because the mean ELF concentration of CAM was ≧8 μg/mL, the individual ELF concentration of CAM in some subjects was less than this target value. Moreover, clinical outcomes were not examined. Based on the decreased concentrations of CAM (0.36–0.80 μg/mL) in this study, it seemed likely that the ELF concentrations of CAM in some subjects who presented poor CAM permeability into the ELF were insufficient and resulted in unfavorable clinical outcomes.

Kobashi and colleagues [31] compared the clinical outcomes of subjects with pulmonary MAC disease who received CAM 400–600 mg and 800 mg. According to their results, the bacilli negative conversion rate for the treatments was 69 and 84 %, respectively. Koga and colleagues [32] reported that the serum CAM concentrations in patients with chronic respiratory infection who received CAM 150–200 mg/day were 1.31–3.45 μg/mL at 1–3 h after administration. Our results (0.36–0.80 μg/mL) after day 15 were lower than those previously reported, probably due to maximum induction of CYP3A4. In some countries, 1000 mg/day of CAM have been commonly administered to pulmonary MAC disease patients [33] and our findings suggest that increasing the dose of CAM over the standard treatment may improve the clinical outcomes. Kawai [34] insisted that the dosage of prednisolone should be doubled to maintain efficacy when used in conjunction with RFP. Matsui and colleagues [35] proposed that, when administered in conjunction with RFP, it was appropriate to increase the dosage of cyclosporine by approximately 3 times within the first 2 weeks, and that long-term frequent measurements of trough level and dose adjustment were required. Based on these reports, it seems likely that the unsatisfactory clinical outcomes in MAC may be attributed to continuously low serum concentrations of CAM and it may be beneficial to reassess the maximum dose of CAM in Japan (800 mg/day), and introduce stepwise administration of CAM for the treatment of pulmonary MAC disease.

Our study has several limitations. First, this study was conducted at a single center. Therefore, the number of subjects was limited. Especially, there were only four subjects who could be followed for 1 year. Secondly, all subjects were outpatients and the severity was mild. Finally, blood samples were collected only after two hours of drug administration on each consultation day. Although more subject data will be needed to verify the correlation between serum CAM concentration and clinical outcomes, we believe that long-term monitoring of CAM is beneficial for therapeutic drug treatment of patients with unsatisfactory clinical outcomes. Increasing the dosage of CAM may improve the outcomes for patients with a poor clinical response to long-term treatment due to low serum concentrations of drug. It may also be useful to decrease the dosage of RFP to a point where efficacy is retained but there is less influence on the induction of CYP3A4. This study provides the first step in the design of a new plan for drug administration appropriate for the treatment of pulmonary MAC disease.

## Conclusions

Our study demonstrated consistently low levels of serum CAM due to RFP-mediated induction of CYP3A4 in pulmonary MAC disease patients treated with a standard combination of CAM and RFP. These continuously low concentrations of CAM are suggested to be responsible for unsatisfactory clinical outcomes of this disease.
